# 399. Epidemiology of Laboratory-identified Late-onset SARS-CoV-2 Positivity in Two Large, Urban, Acute-Care Hospitals: Implications for Surveillance of Hospital-Acquired COVID-19

**DOI:** 10.1093/ofid/ofab466.600

**Published:** 2021-12-04

**Authors:** William Trick, Michael Y Lin, Sharon F Welbel, Onfofre T Donceras, Huiyuan Zhang, Marion Tseng, Carlos Santos

**Affiliations:** 1 Cook County Health and Rush University Medical Center, Chicago, IL; 2 Rush University Medical Center, Chicago, IL; 3 Rush Presbyterian Hosptial, Skokie, IL; 4 John H. Stroger Hospital of Cook County, Chicago, IL; 5 Cook County Health, Chicago, IL; 6 MRAIA, Chicago, IL

## Abstract

**Background:**

Laboratory identification (Lab-ID) of late-onset SARS-CoV-2 positive tests during a hospital stay is a potential public health surveillance approach for hospital-acquired COVID-19. However, prolonged RNA fragment shedding and intermittent detection of SARS-CoV-2 virus via PCR testing among infected patients may hamper interpretation of laboratory-identified events. We aimed to describe the epidemiology of late-onset SARS-CoV-2 laboratory events using clinical criteria, to evaluate the feasibility of a Lab-ID approach to detection of nosocomial SARS-COV-2 infection.

**Methods:**

We evaluated all SARS-CoV-2 RT-PCR positive results recovered from patients at two acute-care hospitals in Chicago, IL, during March 1 — November 30, 2020. Each hospital maintained stringent infection control policies through-out the study period. Through chart review (WT & CS), we categorized all initial SARS-CoV-2 positive tests collected > Hospital Day 5 (defined as ‘late-onset’ based on the 5-day mean incubation period for COVID-19) into the following clinical categories: Community Acquired; Unlikely Hospital Acquired; Possible Hospital Acquired; and Probable Hospital Acquired. Categorizations were made using hospital day, symptoms, alternative diagnoses, and clinical notes (Figure 1).

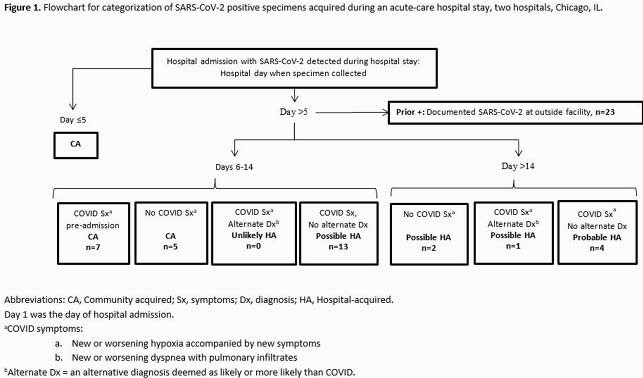

**Results:**

Of 2,671 SARS-CoV-2-positive patients, most positive tests (n=2,551; 96%) were recovered pre-admit or by Hospital Day 2; first positive tests were uncommon during Hospital Days 6 to 14 (n=40; 1.5%); and rare after Hospital Day 14 (n=15; 0.6%). By chart review, of the 55 late-onset records reviewed, categorizations in descending order were: Prior positive at outside facility (n=23); Possible Hospital Acquired (n=16); Community Acquired (n=12); Probable Hospital Acquired (n=4). Less than half of the late-onset cases were categorized as a possible or probable hospital acquisition (Figure 2).

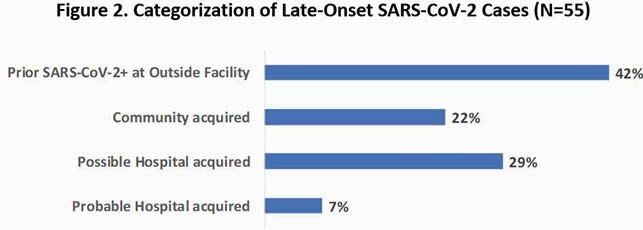

**Conclusion:**

Hospital-acquired SARS-CoV-2 infection was uncommon. Most late-onset episodes of SARS-CoV-2 were explained by detection at an outside healthcare facility or by delayed diagnosis of patients with symptoms at time of presentation. A Lab-ID approach to nosocomial COVID-19 surveillance would potentially misclassify a substantial number of patients.

**Disclosures:**

**All Authors**: No reported disclosures

